# The Office and Eccentricities of the Dental Pulp

**Published:** 1896-01

**Authors:** D. D. Smith

**Affiliations:** Philadelphia, Pa.


### The Office and Eccentricities of the Dental Pulp.

BY D. D. SMITH, PHILADELPHIA, PA.

Abstract of paper read at American Dental Association, August, 1895.

It is well known that the different formations which make up the body of the tooth receive life and nourishment from two distinct sources; from the pulp and from the pericementum of the root. The pulp is the central figure; the important factor of every tooth. To it is committed the care of the newly erupted tooth and its office work is to readjust, recalcify, consolidate, strengthen and sustain the enamel and dentine.

As a rule the earlier a tooth is developed the more readily it yields to decay, and the more time taken for calcification before eruption, the greater the resistance to decay manifested on the part of the tooth. * * *

The law of *use* governing tooth consolidation, widely known and rigidly observed among patients, would probably do more to arrest the extensive decay now prevalent, between eight and fifteen years of age, than dentistry can ever accomplish by mechanical methods.

Too much importance has been and is attached to inheritance as determining the character of the teeth. Standing in advance of inheritance, for children, is *use* of the teeth in mastication. An effort should be made to save the first permanent molar. Devitalization of the pulp carries with it a more or less rapid retrogressive change in the quality of tooth material, and that without power to arrest it. Fillings may prolong the existence of the tooth, as such, but with the arrest of vitality in the pulp there is cessation of all vital sustaining action, which hitherto assisted in its preservation, and not only so, the imperfectly calcified enamel and dentine already built into the tooth, is now in contact with devitalized connective tissue, which, in the imperfectly consolidated tooth, becomes itself probably a source of disintegration, and assists in its destruction. What has been said of the first permanent molar may also be said of all other of the young permanent teeth. Later in life we do not find teeth which have been decay resisting for fifty or sixty years, changing in structure, assuming a state of partial decalcification, returning again to the conditions of childhood, as evidenced by their yielding more readily to decay.

The teeth have softened and if refilled with gold, in a comparatively short time they begin to darken around the fillings. * * *

Now, what has brought about this change? Is it not the pulp after a period of practical inactivity again at work but now transforming the compact material, which it placed in the tooth, in a condition wherein it yields much more readily to decay? * * *

As the tooth is when the pulp is destroyed, so it must remain except as to those changes which take place through the gradual disintegration of the internal structure of all pulpless teeth. * * *

Pyorrhea alveolaris is seldom or never found in connection with young teeth; it seems to be emphatically a disease of adult life; generally middle life. It is never found in connection with devitalized teeth, where devitalization preceded the manifestation of the disease.

